# Hepatoprotective effect of ethanolic extract of *Trichosanthes lobata* on paracetamol-induced liver toxicity in rats

**DOI:** 10.1186/1749-8546-7-12

**Published:** 2012-05-18

**Authors:** Aiyalu Rajasekaran, Muthusamy Periyasamy

**Affiliations:** 1 Department of Pharmaceutical Chemistry, KMCH College of Pharmacy, Kovai Estate, Kalapatti Road, Coimbatore, Tamilnadu, 641 048, India; 2 Department of Pharmacology, KMCH College of Pharmacy, Kovai Estate, Kalapatti Road, Coimbatore, Tamilnadu, 641 048, India

## Abstract

**Background:**

*Trichosanthes lobata* (family cucurbitaceae) is used to treat malarial fever and liver disorders. This study aims to investigate possible hepatoprotective activities of ethanolic extract of *Trichosanthes lobata* against paracetamol-induced hepatotoxicity.

**Methods:**

Hepatotoxicity was induced in Wistar male rats by oral administration, 2 g/kg body weight on 7th day after the administration of ethanolic extract of *Trichosanthes lobata* and silymarin (100 mg/kg). Ethanolic extract of *Trichosanthes lobata* was administered orally at doses of 200 mg/kg and 400 mg/kg body weight daily for 7 days. Several serum markers, aspartate transaminase, alanine transaminase, alkaline phosphatase, bilirubin, total protein was measured to assess the effect of the extract on paracetamol (acetaminophen)-induced hepatic damage. The study included histopathological examination of liver sections.

**Results:**

Blood samples from rats treated with ethanolic extract of *Trichosanthes lobata* (200 mg/kg body weight and 400 mg/kg body weight) had significant reductions in serum markers in paracetamol administered animals, indicating the effect of the extract in restoring the normal functional ability of hepatocytes. Silymarin (100 mg/kg, p.o.) was used as a reference drug.

**Conclusion:**

The ethanolic extract of *Trichosanthes lobata* exhibits protective effects against paracetamol‒induced hepatotoxicity.

## Background

Hepatotoxicity is a common cause of severe metabolic disorders and even death [1]. Flavonoids exhibit vasoprotective, anti-inflammatory, anti-allergic, antimicrobial, antioxidant, hepatoprotective, anti-osteoporotic, and anti-neoplastic properties [2]. *Trichosanthes lobata* (wild snake gourd, family cucurbitaceae), *Trichosanthes dioica*[3,4]*Trichosanthes cucumerina*[5-12], and *Trichosanthes kirilowii*[13] contain carbohydrates, glycosides, flavonoids, tannins, proteins, steroids, and saponins and *Trichosanthes lobata* is used for malarial fever and liver disorders [14,15].

Paracetamol (acetaminophen) is widely used as an antipyretic and analgesic, and it produces acute liver damage if administrated in excess [16,17]. Paracetamol is mainly metabolized in the liver to excretable glucuronide and sulphate conjugates [18,19]. However, the hepatotoxicity of paracetamol has been attributed to the formation of toxic metabolites when part of it is activated by hepatic cytochrome P‒450 [20] to form the highly reactive metabolite N‒acetyl‒P‒benzoquinone imine (NAPQI) [21]. NAPQI covalently binds to cysteine groups on proteins to form 3-(cystein-S-yl) acetaminophen adducts [22]. The glutathione protects hepatocytes by combining with the reactive metabolite of paracetamol, thus preventing covalent binding to liver proteins [23].

The experimental demonstration of the hepatoprotective activities is lacking. This study aims to investigate possible hepatoprotective properties of *Trichosanthes lobata*.

## Methods

### Chemicals

Paracetamol (acetaminophen) was purchased from S.D. Fine Chemicals, Ltd. (India). A gift sample of Silymarin was provided by Ranbaxy (Devas, India), and standard assay kits of aspartate transaminase (AST), alanine transaminase (ALT), alkaline phosphatase (ALP), bilirubin and total protein was obtained from Jain Scientific Industries (Moradabad, India). All other reagents were of analytical grade.

### Plant materials

The plant *Trichosanthes lobata* was collected in Malapuram district, Kerala. The plant was authenticated (BSI/SRC/5/23/2010-11/Tech-1833 dt 4 February 2011) by Dr. Govindappa.Venkatesa Sundara Murthy, of the Botanical Survey of India, Coimbatore. Plants were authenticated with the existing herbarium specimens in central national herbarium (Botanical Survey of India, Coimbatore, India), after treatment of the plants with saturated solution of mercuric chloride and ethyl alcohol [24]. A voucher specimen (KMCH/COG/Tl/2011/02) was deposited at Department of Pharmacognosy, KMCH College of Pharmacy, Coimbatore, for future reference.

### Preparation of extract

Extract of dried powdered *Trichosanthes lobata* was obtained with 70% ethanol (Merck, Germany) in a Soxhlet apparatus at 68°C for 72 h. The extract was filtered on Whatman No. 1 filter paper (Scientific Furnishings, Ltd., Chichester, UK) and concentrated using a rotary vacuum evaporator (N-1001 T-WD, Eyele, Japan) at 40°C - 45°C (0.9% w/w).

### Phytochemical screening

Phytochemical screening was carried out by standard procedures, as described by Kokate [25] and Harborne [26] (Table 1). 

**Table 1:** **Preliminary phytochemical screening of ethanolic extract of *T. lobata***

**Constituent**	**Ethanolic Extract**
Triterpenes	-
Steroids	+
Carbohydrates	+
Tannins	+
Flavonoids	+
Alkaloids	-
Glycosides	+
Saponins	+
Protein	+

### Thin-layer chromatography

Thin-layer chromatography (TLC) for ethanolic extract of *Trichosanthes lobata* was performed on precoated silica gel 60 GF_254_ (MERCK, Germany) using mobile phase n-hexane: ethyl acetate (Qualigens, India) (7:3) and visualized by UV light after treatment with anisaldehyde-sulphuric acid reagent (Qualigens, India).

### Experimental animals

Swiss female mice (20–25 g) and albino adult Wister male rats (150–200 g) were obtained from the animal house of KMCH College of Pharmacy, Tamilnadu, India. The study protocol was approved by the institutional animal ethics committee, Committee for the Purpose of Control and Supervision on Experimental Animals (CPCSEA), New Delhi, India, as per approval no. 509/01/C/CPCSEA dt, 10 January, 2009. Both rats and mice were housed in plastic cages (47 × 34 × 18 cm) in an air-conditioned environment, with 10 mice per cage or 6 rats per cage. The floor of the cages was lined with saw dust, which was replaced every 48 h. Both rats and mice were fed with standard pellet diet (Kamadenu Enterprises, *Bangalore, India), and they* had free access to water*.*

### Acute toxicity test

Acute oral toxicity was determined according to method described by Litchfield [27]. Female mice were divided into 8 groups of 6 animals each. The control group received 0.5 mL of 0.5% w/v sodium carboxymethyl cellulose (Qualigens, India) orally. The other groups received 100, 200, 400, 800, 1000, 2000, and 3000 mg/kg body weight ethanol extract of *Trichosanthes lobata* in 0.5% sodium carboxymethyl cellulose orally. Immediately after dosing, the mice were continuously observed for at least 4 h, and occasionally up to 6 h. They were then observed for up to 14 days (frequency of 12 h/day) for signs of toxicity and mortality.

### Hepatoprotective activities

Paracetamol induced hepatotoxicity model was adopted for the study [28]. The rats were divided into 5 groups of 6 animals each. Group I served as a control and received normal saline, 5 mL/kg body weight, daily for 7 days. Group II constituted the hepatotoxic group and were treated similarly to group I. Group IV and Group V received ethanolic extract (200 and 400 mg/kg body weight per day, respectively) suspended in 0.5% sodium carboxymethylcellulose for 7 days. Group III received the reference drug, silymarin (100 mg/kg body weight daily) for 7 days.

On the 7th day, paracetamol suspension was given orally, 2 g/kg body weight, to all the rats except those in Group I. At the end of the experimental period, the rats were fasted overnight and sacrificed by ether. Blood and liver samples were collected for biochemical and histological studies.

### Histopathological studies

Paraffin sections (7 μm thick) of buffered formalin–fixed liver samples were stained (nuclei in blue and cytoplasm in pink) with hematoxylin-eosin [29] to identify the histological changes under the microscope (Vision micro systems, India).

#### Biochemical studies

Blood was obtained from all animals by puncturing the retro-orbital plexus. Blood samples were allowed to clot for 45 min at room temperature. Serum was separated by centrifugation [Model No.LAC 10370, Remi, India] at 2.5 x *g* at 30°C for 15 min and assayed for AST, ALT, ALP [30], bilirubin [31] and total protein [32], as shown in Table 2. 

**Table 2:** Serum biochemical parameters

** Parameter**				**Groups (n = 6 for each group)**				
	**Control (Group I)**	**Paracetamol**	**Paracetamol +**	**Ethanolic Extract of *Trichosanthes lobata***		
		**2 g/kg body weight (Group II)**	**silymarin 100 mg/kg body weight (Group III)**	**200 mg/kg body weight (Group IV)**	**400 mg/kg body weight (Group V)**	
AST (U/l)	37.67 (3.803)	127.3 (25.51)^a^	30.83 (7.705)^a^	32.00 (6.899)^a^	18.50 (9.182)^a^
ALT (U/l)	21.67 (1.966)	146.3 (32.38)^a^	68.17 (22.44)^a^	64.00 (9.011)^b^	37.50 (6.686)^ns^
ALP (U/l)	187.2 (20.23)	312.0 (62.24)^a^	204.5 (55.56)^a^	210.7 (30.75)^b^	171.80 (19.16)^a^
Bilurubin (mg/dl)	0.63 (0.2160)	1.10 (0.2191)^b^	0.40 (0.2608)^a^	0.35 (0.1871)^a^	0.23 (0.1033)^a^
Total protein (g/dl)	1.80 (0.4011)	2.03(0.0811)^ns^	1.83 (0.3084)^ns^	1.77 (0.3830)^ns^	2.11 (0.3147)^ns^

a, *P* < 0.001; b, *P* < 0.01; ns : non significant.

Values are expressed as mean (standard deviation; SD). Statistical significance was calculated with ANOVA followed by Dunnett test comparing treated group with paracetamol group.

### Statistical analysis

For determination of significant inter-group differences of each parameter one-way analysis of variance (ANOVA) was carried out. Dunnet test was used for individual comparisons after significant ANOVA results. The differences with *P* < 0.05 were considered statistically significant. GraphPad prism 4 software (GraphPad Software, Inc**.** California, USA) was used for the statistical analysis.

## Results

### Phytochemical investigation and TLC study

Phytochemical screening of the ethanolic extract of *Trichosanthes lobata* confirmed the presence of proteins, steroids, tannins, carbohydrates, glycosides, saponins, and flavonoids.

The spots obtained after TLC development revealed that the ethanolic extract possesses flavonoids, saponins, and tannins.

### Acute toxicity test

The ethanolic extract of *Trichosanthes lobata* did not result in any mortality of mice up to the dose of 3000 mg/kg body weight. Hence, doses of 200 and 400 mg/kg body weight were selected.

### Histopathological studies

**Figure 1:**
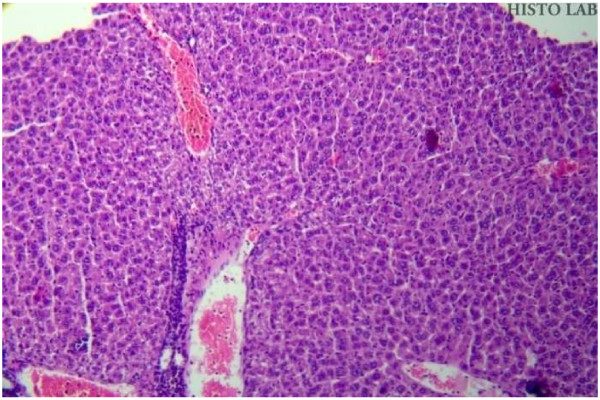
Liver section of normal rats (Group I) showing moderate sinusoidal and central vein dilatation and congestion with prominent nucleus.

**Figure 2:**
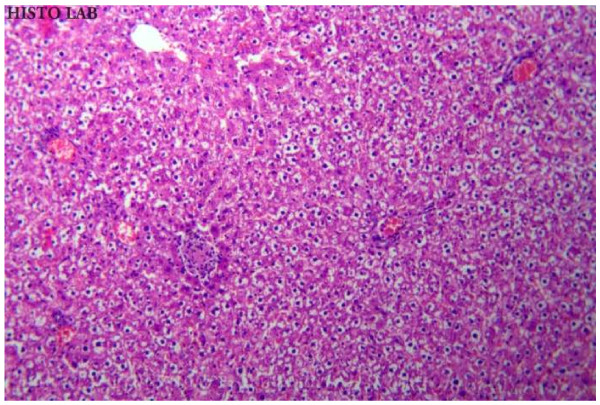
Liver section of paracetamol (2 g/kg, treated rats (Group II) showing hydropic degeneration and focal necrosis.

**Figure 3:**
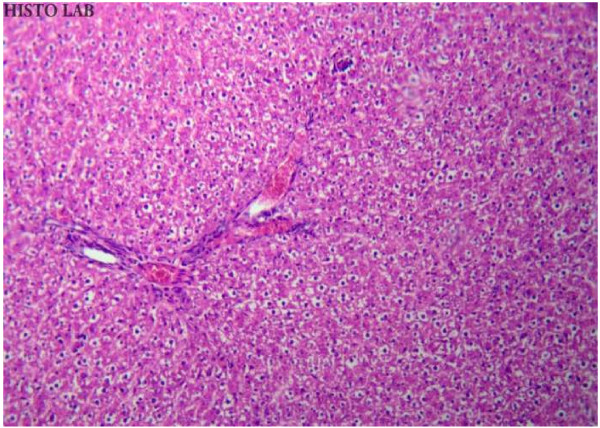
Liver section of rats treated with paracetamol (2 g/kg, p.o) + ethanolic extract (200 mg/kg, p.o) × 14 days (Group IV) hydrophobic lesions with congestion and mild signs of necrosis.

**Figure 4:**
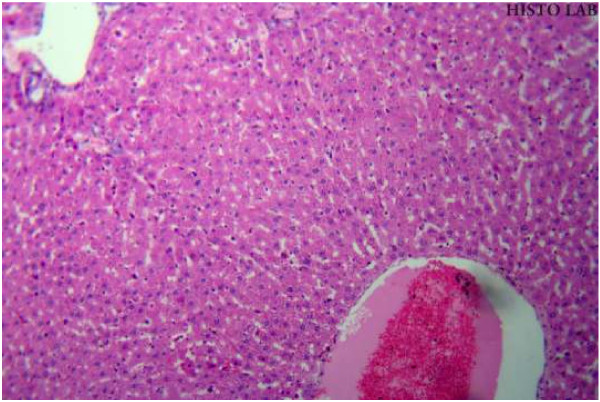
Liver section of paracetamol (2 g/kg, p.o) + ethanolic extract (400 mg/kg, p.o) × 14 days (Group V) showing mild congestion with no signs of necrosis.

**Figure 5:**
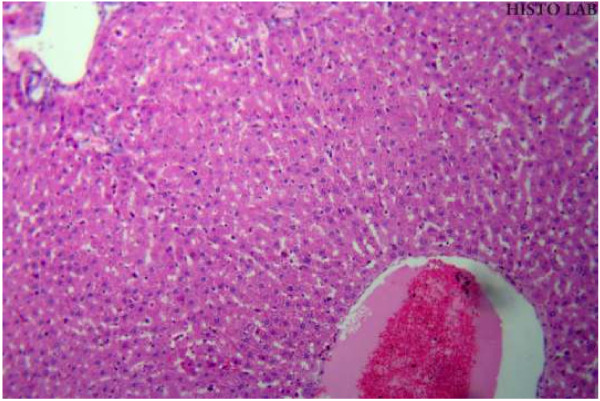
Liver section of rats treated with paracetamol (2 g/kg, p.o) + silymarin (100 mg/kg, p.o) × 14 days (Group III) showing normal histological appearance IV) showing with no signs of necrosis.

Histopathological studies of rat liver tissue from the control group (Group I) showed normal hepatic cells with central vein and sinusoidal dilation (Figure 1). In the paracetamol group (Group II), severe hepatotoxicity was observed in the form of severe necrosis and disappearance of nuclei (Figure 2). Histopathological analysis showed that the pathological lesions caused by paracetamol were very minimal in groups pretreated with ethanolic extract of *Trichosanthes lobata* (Group IV and V). Normal hepatocytes with regenerating hepatocytes and mild inflammation in the portal area were observed in groups IV and V, treated with ethanolic extract of *Trichosanthes lobata*, 200 and 400 mg/kg body weight, respectively (Figures 3 and 4). Liver tissue from paracetamol + silymarin group (Group III) had normal hepatic cells with portal vein and portal artery (Figure 5).

### Biochemical studies

The effects of ethanolic extract of *Trichosanthes lobata* on AST, ALT, ALP, bilirubin, and total protein levels in rats with paracetamol-induced liver damage were summarized in Table 2. Administration of paracetamol (2 g/kg body weight, orally) after 18 h resulted in a significant (*P* < 0.05) elevation of hepatospecific serum markers such as AST, ALT, ALP, bilirubin, and total protein in the paracetamol group (Group II) in comparison with the control group (Group I). On administration of ethanolic extract of *Trichosanthes lobata* (Groups IV and V) and paractamol + silymarin group (Group III), the serum markers were restored to the normal levels.

## Discussion

Histopathological studies of rats administered paracetamol showed severe necrosis and disappearance of nuclei. This could be due to the formation of highly reactive metabolites (*e.g.* NAPQI), because of excessive administration of paracetamol. All these histopathological changes were significantly reduced in rats treated with ethanolic extract of *Trichosanthes lobata*.

The study of serum markers such as AST, ALT, ALP, and bilirubin, and total protein has been found to be of great value of assess to clinical and experimental liver damage [33]. In the present investigation, the rats suffered significant hepatic damage from treatment with paracetamol, as indicated by elevated levels of serum markers (Table 2). A rise in AST is usually accompanied by an increase in ALT, which plays a vital role in the conversion of amino acids to keto acids [34]. Pretreatment with ethanolic extract of *Trichosanthes lobata*, both at 200 mg/kg body weight and 400 mg/kg body weight, significantly attenuated elevated levels of serum markers. This suggests that ethanolic extract of *Trichosanthes lobata* conditions the hepatocytes so as to protect the integrity of the membrane from paracetamol-induced leakage of serum markers into circulation. These changes can be considered a functional improvement of hepatocytes and may be caused by accelerated regeneration of parenchyma cells. Serum ALP and bilirubin are related to hepatic cell damage [28]. Increase in serum ALP is due to increased synthesis in the presence of increasing biliary pressure [35]. The decrease in the levels of ALP and bilirubin may be due to the presence of flavonoids and their antioxidant effects which may protect the hepatic cell damage induced by paracetamol.

A potential of hepatoprotective property underlying *Trichosanthes lobata* may be attributed to the anti-oxidative constituents. The plants most commonly used to treat liver disorders are *Curcuma longa* (turmeric)*, Glycyrrhiza glabra* (licorice), and *Camellia sinensis* (green tea), and they are all reported to be hepatoprotective due to the powerful anti-oxidative properties [36-39]. Also, the antioxidant properties of *Trichosanthes cucumerina* are attributed to flavonoids, carotenoids, lycopene, phenolics, and β-carotene [40]. The presence of flavonoids, saponins, and tannins in ethanolic extract of *Trichosanthes lobata* was confirmed by phytochemical analysis and TLC, and these compounds are reported to have antioxidant properties [41].

## Conclusions

The ethanolic extract of *Trichosanthes lobata* exhibits protective activities against paracetamol-induced hepatotoxicity.

## Competing interests

The authors declare that they have no competing interests.

## Author’s contributions

AR performed extraction, phytochemical evaluation, wrote and revised the manuscript. MP conducted pharmacological and toxicological studies, and performed statistical analysis. All authors read and approved the final manuscript.

## References

[B1] Patel RK Patel MM Patel MP Kanzaria NR Vaghela KR Patel NJ Hepatoprotective activity of *Moringa oleifera* Lam. Fruit on isolated rat hepatocytes Pharmacogn Mag 2008 4 118 123

[B2] Havsteen B Flavonoids, a class of natural products of high pharmacological potency Biochem Pharmacol 1983 32 1141 1148 10.1016/0006-2952(83)90262-9 6342623

[B3] Bhattacharya S Kanti Halder P Antibacterial activity of Trichosanthes diocia root Global J pharmacol 2010 4 3 122 126

[B4] Badrul Alam M Sarowar Hossain M Sultana Chowdary N Asadujjaman Ronok Zahan Monirul Islam Ehsanul Haque Mazumder Ekramul Haque Anwarul Isalm Antioxidant, anti-inflammatory and anti-pyretic activities of *Trichosanthes diocia* Roxbfruits J Pharmacol Toxicol 2011 6 5 440 453 10.3923/jpt.2011.440.453

[B5] Kolte RM Bisan VV Jangde CR Bhalerao AA Anti-inflammatory activity of root tubers of *Trichosanthes cucumerina* in mouse’s hind paw oedema induced by carrageenin Indian J Indigeneous Medicines 1997 18 2 117 121

[B6] Kongtun S Jiratchariyakul W Mongkarndi P Theppeang K Sethajintanin I Jaridasem S Frahm AW Thai: Cytotoxic properties of root extract and fruit juice of *Tricosanthes cucumerina* J Phytopharm 1999 6 2 1 9

[B7] Kar A Choudhury BK Bandyopadhyay NG Comparative evaluation of hypoglycaemic activity of some Indian plants in alloxan diabetes rats Journal Ethnopharmacol 2003 84 1 105 108 10.1016/S0378-8741(02)00144-7 12499084

[B8] Arawwawala M Thabrew I Arambewela L Antidiabetic activity of *Trichosanthes cucumerina* in normal and streptozotocin–induced diabetic rats Int J Biol Chem Sci 2009 3 2 56 10.3923/ijbc.2009.56.70

[B9] Kirana H Srinivasan B *Tricosanthes cucumerina* improves glucose tolerance and tissue glycogen in non insulin dependent diabetes mellitus induced rats Indian J Pharmacol 2008 3 345 348 10.4103/0253-7613.42301 PMC2792599 20040935

[B10] Sathesh KS Ravi KB Krishna MG Hepatoprotective effect of *Tricosanthes cucumerina* L on carbon tetrachloride induced liver damage in rats J Ethnopharmacol 2009 123 2 347 350 10.1016/j.jep.2009.02.023 19429383

[B11] Devendra N Vijay KB Mala S Effect of ethanol extract of whole plant of Tricosanthes cucumerina Var. Cucumerina on gonadotropins, ovarian follicular kinetics and estrous cycle for screening of anti fertility activity in albino rats Int J Morphol 2009 27 1 173 182

[B12] Arawwawala LD Thabrew MI Arambewela LS Gastroprotective activity of *Trichosanthes cucumerina* in rats J Ethnopharmacol 2010 3 750 754 19963056 10.1016/j.jep.2009.11.026

[B13] Leung KN Yeung HW Leung SO The immunomodulatory and antitumour activities of trichosanthin-an abortificient protein isolated from Tian-hua-fen (*Trichosanthes kiwilowii*) Asian Pacific J Allergy Immunol 1986 4 111 120 3492210

[B14] Karuppusamy S Medicinal plants used by Paliyan tribes of sirumalai hills of south India Nat Prod Rad 2007 6 5 436 442

[B15] Jalali Far MA Alinejadi M Gholam Abas K Saki N Negravi S Liver Function Tests and Demography Profiles of HBV-DNA Positive Patients Referred to Naft Great Hospital in 2009-2010. The 21st Conference of the Asian Pacific Association for the Study of the Liver Hepatol Int 2011 5 3 55821484580

[B16] Black M Acetaminophen hepatotoxicity Annu Rev Med 1984 35 577 593 10.1146/annurev.me.35.020184.003045 6372672

[B17] Davidson DG Eastham WN Acute liver necrosis following over dose of paracetamol Br Med J 1966 5512 497 499 5913083 10.1136/bmj.2.5512.497 PMC1943529

[B18] Nanji AA Jokelainen K Fotouhinia M Rahemutulla A Thomass P Tipoe LG Su GL Dannenberg AJ Increased severity of alcoholic liver injury in female rats: role of oxidative stress, endotoxin and chemokines Am J Physiol 2002 281 1348 1356 10.1152/ajpgi.2001.281.6.G1348 11705739

[B19] Jollow DJ Thorgeirsson SS Potter WZ Hashimoto M Mitchell JR Acetaminophen induced hepatic necrosis VI. Metabolic disposition of toxic and non‒toxic doses of acetaminophen Pharmacology 1974 12 251 271 10.1159/000136547 4449889

[B20] Wong LT Whitehouse LW Solemonraj G Paul CJ Pathways of Acetaminophen conjugate in the mouse Toxicity Lett 1981 9 145 151 10.1016/0378-4274(81)90031-X 7302986

[B21] Savides MC Oehne FW Acetaminophen and its toxicity J App Toxicol 1983 3 95 111 10.1016/S0272-0590(83)80062-1 6886301

[B22] Vermeulen NPE Bessems JGM Van de Streat R Molecular aspects of paracetamol‒induced hepatotoxicity and its mechanism based prevention Drug Metab Rev 1992 24 367 407 10.3109/03602539208996298 1628537

[B23] Tirmenstein MA Nelson SP Sub cellular binding and effects on calcium homeostasis produced by acetaminophen and a non-hepatotoxic region isomer 3-hydroxyacetanilide in mouse liver J Biol Chem 1989 264 9814 9819 2524496

[B24] Jain SK Rao RR A handbook of field and herbarium methods 1977 New Delhi: Today & Tomorrow’s printers and publishers

[B25] Kokate CK Practical Pharmacognosy 1986 1 New Delhi: Vallabh Prakashan 111

[B26] Harbone JB Methods of extraction and isolation Phytochemical Methods 1998 London: Chapman and Hall 60 66

[B27] Litchfield JT Wilcoxon F A simplified method for evaluating dose effect experiments J Pharmacol 1943 96 99 113 18152921

[B28] Dash Deepak K Yeligar Veerendra C Nayak Siva S Tirtha Ghosh Rajalingam D Pinaki Sengupta Maiti Bhim C Maity Tapan K Evaluation of hepatoprotective and antioxidant activity of *Ichnocarpus frutescens* (Linn.) R.Br. on paracetamol-induced hepatotoxicity in rats Trop J Pharm Res 2007 6 3 755 765

[B29] Gornall AG Bardwill CJ David MM Determination of serum proteins by means of the biuret reaction J Biol Chem 1949 177 751 756 18110453

[B30] Retimen S Frankel SA Colorimetric method for determination of serum glutamic oxaloacetic and glutamic pyruvate transaminases Am J Clin Pathol 1957 28 56 63 13458125 10.1093/ajcp/28.1.56

[B31] King EJ Armstrong AR A convenient method for determining of Serum and bile phosphatase activity J Canad Med Assoc 1934 31 376 381 PMC403559 20319659

[B32] Malloy HT Evelyn KA The determination of bilirubin with the photometric colorimeter J Biol Chem 1937 119 481 490

[B33] Moore M Thor H Moore G Nelson S Moldeus P Correnius S The toxicity of acetaminophen and N‒acetyl P-benzoquinone imine in isolated hepatocytes is associated with thio depletion and increased cytosolic Ca^2+^ J Biol Chem 1985 260 13035 13040 2932433

[B34] Vaishwanar I Kowale CN Effect of two ayurvedic drugs Shilajeet and Eclinol on changes in liver and serum lipids produced by carbon tetrachloride Ind J Exp Biol 1976 14 58 61 955691

[B35] Sallie R Tredger JM William Drugs and the liver Biopharm Drug Dispos 1999 12 251 259 1873506 10.1002/bdd.2510120403

[B36] Donatus IA Sardjoko Vermeulen NP Cytotoxic and cytoprotective activities of curcumin. Effects on paracetamol induced cytotoxicity, lipid peroxidation and glutathione depletion in rat hepatocytes Biochem Pharmacol 1990 39 1869 1875 10.1016/0006-2952(90)90603-I 2353930

[B37] Soni KB Lahiri M Chackradeo P Protective effect of food addities on aflatoxin-induced mutagenicity and hepatocarcinogenicity Cancer Lett 1992 115 115 121 9149115 10.1016/s0304-3835(97)04710-1

[B38] Wang GS Han ZW The protective action of Glycyrrhiza flavonoids against carbon tetrachloride hepatotoxicity in mice Yao Hsueh Hsueh Pao 1993 28 572 576 8285064

[B39] Miyagawa C Wu C Kennedy DO Protective effect of green tea extract and tea polyphenols against cytotoxicity of 1,4-naphthoquinone in isolated rat hepatocytes Biosci Biotechnol Biochem 1997 61 1901 1905 10.1271/bbb.61.1901 9404069

[B40] Lavelli V Peri C Rizzolo A Antioxidant activity of tomato products as studied by model reactions using xanthine oxidase, myeloperoxidase, and copper induced lipid peroxidation J Agric Food Chem 2000 48 1442 1448 10.1021/jf990782j 10820040

[B41] Shankar MB Parikh JR Geetha M Mehta RS Saluja AK Hepatoprotective activity of benzopyrone from *Tephrosia purpurea* Pers J Nat Rem 2005 5 2 115 120

